# The Elevated Endogenous Reactive Oxygen Species Contribute to the Sensitivity of the Amphotericin B-Resistant Isolate of *Aspergillus flavus* to Triazoles and Echinocandins

**DOI:** 10.3389/fmicb.2021.680749

**Published:** 2021-08-03

**Authors:** Tianyu Liang, Wei Chen, Xinyu Yang, Qiqi Wang, Zhe Wan, Ruoyu Li, Wei Liu

**Affiliations:** Department of Dermatology and Venerology, Peking University First Hospital, National Clinical Research Center for Skin and Immune Diseases, Research Center for Medical Mycology, Peking University, Beijing Key Laboratory of Molecular Diagnosis on Dermatoses, Beijing, China

**Keywords:** *Aspergillus flavus*, amphotericin B, reactive oxygen species, triazoles, echinocandins

## Abstract

*Aspergillus flavus* has been frequently reported as the second cause of invasive aspergillosis (IA), as well as the leading cause in certain tropical countries. Amphotericin B (AMB) is a clinically important therapy option for a range of invasive fungal infections including invasive aspergillosis, and *in vitro* resistance to AMB was associated with poor outcomes in IA patients treated with AMB. Compared with the AMB-susceptible isolates of *A. terreus*, the AMB-resistant isolates of *A. terreus* showed a lower level of AMB-induced endogenous reactive oxygen species (ROS), which was an important cause of AMB resistance. In this study, we obtained one AMB-resistant isolate of *A. flavus*, with an AMB MIC of 32 μg/mL, which was sensitive to triazoles and echinocandins. This isolate presented elevated endogenous ROS levels, which strongly suggested that no contribution of decreased AMB-induced endogenous ROS for AMB-resistance, opposite to those observed in *A. terreus*. Further, we confirmed that the elevated endogenous ROS contributed to the sensitivity of the AMB-resistant *A. flavus* isolate to triazoles and echinocandins. Further investigation is needed to elucidate the causes of elevated endogenous ROS and the resistance mechanism to AMB in *A. flavus*.

## Introduction

Invasive aspergillosis (IA) is an important opportunistic fungal infection caused by *Aspergillus* with high mortality rates. Over the past few decades, the incidence of IA has been rising with the increasing number of immunosuppressed patients (Brown et al., 2012). *Aspergillus flavus* has been frequently reported as the second leading cause of IA, as well as the leading cause in certain tropical countries (Rudramurthy et al., 2019).

At present, there are three main classes of antifungal drugs used for the treatment of IA (Perlin et al., 2017): (i) polyenes, such as amphotericin B (AMB); (ii) triazoles, such as itraconazole (ITC), voriconazole (VRC) and posaconazole (POS); and (iii) echinocandins, such as caspofungin (CAS) and micafungin (MFG). Among them, AMB stood out due to its broad activity spectrum and less likely developed drug resistance. AMB has been a clinically important therapy option for a range of invasive fungal diseases, including IA, since it was first approved in the 1950s (Perlin et al., 2017). Although dose-dependent toxic side effects, such as nephrotoxicity, limit the use of AMB, the lipid formulations of AMB with equal antifungal activity were therefore developed to reduce these toxicity issues (Stone et al., 2016; Grazziotin et al., 2018). Although AMB resistance is rare, the pathogenic *A. terreus* is intrinsically resistant to AMB (Vaezi et al., 2018) and the reports of the AMB-resistant *A. fumigatus* and *A. flavus* were also notable (Ashu et al., 2018; Rudramurthy et al., 2019). Moreover, *in vitro* resistance to AMB was associated with poor outcomes in IA patients treated with AMB (Hadrich et al., 2012). Therefore, it is important to elucidate the mechanisms of AMB resistance.

Until now, the mode of antifungal action of AMB has not been well understood, and the mechanisms of AMB resistance also need to be elucidated. In addition to binding to ergosterol directly (Gray et al., 2012) and forming ion channels (Kristanc et al., 2019) thereby disrupting the structural integrity of cell membranes, several studies have highlighted that AMB exert antifungal activity by inducing endogenous reactive oxygen species (ROS) production, therefore resulting in oxidative damage and fungal cell death (Belenky et al., 2013; Mesa-Arango et al., 2014; Shekhova et al., 2017). Studies on *A. terreus*, intrinsic resistance to AMB, revealed that the AMB-resistant clinical isolates of *A. terreus* could handle better with AMB-induced oxidative stress and thus showed a lower level of AMB-induced endogenous ROS, compared with AMB-susceptible clinical isolates of *A. terreus* (Blatzer et al., 2015; Jukic et al., 2017). These studies are important for understanding the mechanisms of resistance to AMB in pathogenic fungi, including *A. flavus*.

In this study, we screened the susceptibility of clinical isolates of *A. flavus* to AMB by using the broth microdilution method according to the Clinical and Laboratory Standards Institute (CLSI) M38-A3 guideline. From 117 clinical isolates of *A. flavus*, we obtained an AMB-resistant *A. flavus* isolate. To investigate the role of ROS in AMB resistance in this isolate, the sensitivity to oxidative stress and endogenous ROS levels with or without exposure to AMB were determined. Meanwhile, the expression level and activities of enzymes involved in ROS detoxification were also investigated. In addition, the endogenous ROS levels induced by triazoles and echinocandins were also measured, and the ROS scavenger N-acetylcysteine (NAC) was used to investigate the effect of ROS levels on *in vitro* antifungal susceptibility in AMB-resistant *A. flavus* isolate.

## Materials and Methods

### Antifungal Susceptibility Testing

Antifungal susceptibility testing by the broth microdilution method was performed according to the recommendations of the CLSI M38-A3 document (Clinical and Laboratory Standards Institute (CLSI)., 2017), and the tested drugs included were ITC, VRC, POS, CAS, MFG (all from Harveybio Gene Technology Co., Ltd., Beijing, China) and AMB (North China Pharmaceutical Co., Ltd., Shijiazhuang, China). Briefly, antifungal drugs were dispensed into 96-well plates at final concentration ranges of 0.0625–32 μg/mL for AMB, 0.0313–16 μg/mL for ITC, VRC, and POS, and 0.008–4 μg/mL for CAS and MFG. All isolates of *A. flavus* were subcultured on potato dextrose agar (PDA) at 35°C for 3 to 7 days to yield good conidiation. Conidia were harvested by slightly scraping the surface of the *A. flavus* colonies with a sterile cotton swab and suspending the colonies in sterile saline solution with 0.05% Tween-20. Heavy particles were allowed to settle for 5 min, after which the upper homogenous suspensions were used as inoculum suspensions. Inoculum suspensions were diluted in RPMI 1640 medium at a final concentration of 1 × 10^4^ CFU/mL, as determined by a hemocytometer, and transferred into 96-well plates containing drug dilutions. The 96-well plates were incubated at 35°C and examined visually for MIC (after 48 h) and MEC (after 24 h) determinations. The MIC endpoints for AMB and triazoles were determined as the lowest drug concentration that resulted in a 100% reduction in growth compared with that of the drug-free controls. The MEC endpoints for echinocandins were determined as the lowest drug concentration that led to the growth of small, rounded, compact hyphal forms compared with the hyphal growth seen in the growth control well.

Antifungal susceptibility testing by E-test was performed according to the manufacturer’s instructions. Briefly, inoculum suspensions at a final concentration of 1 × 10^6^ CFU/mL were inoculated on the entire surface of each 90-mm plate containing 25 mL of RPMI 1640 medium (in the presence or absence of 15 mM NAC) with a sterile cotton swab. The E-test strips (Autobio, Zhengzhou, China) were placed on the center of the plate and incubated at 35°C. The MIC or MEC (for CAS only) was determined from the inhibition ellipse that intersected the scale on the strip after 48 h.

Antifungal susceptibility testing by disk diffusion was performed on non-supplemented Muller-Hinton (NMH) agar refer to the method described previously (Qiao et al., 2007). When necessary, a 15 mM concentration of the antioxidant NAC was dissolved in NMH medium. Disks prepared in-house of AMB (50 μg), ITC (10 μg), VRC (5 μg), POS (5 μg), CAS (5 μg), and MFG (5 μg) were placed onto the surface of the inoculated (the same method as described in the E-test) NMH plate. The plates were incubated at 35°C, and the inhibition zone diameter was determined after 48 h.

### Testing of Sensitivity to Oxidative Stress

Based on the reported studies that AMB-resistant *A. terreus* can handle oxidative stress better, two *A. terreus* clinical isolates with different susceptibilities to AMB, the AMB-susceptible *A. terreus* isolate BMU09523 (MIC = 2 μg/ml) and the AMB-resistant *A. terreus* isolate BMU05143 (MIC = 8 μg/mL) were included for comparison. We tested the sensitivity of *A. flavus* isolates and *A. terreus* isolates to H_2_O_2_ by spot assay. H_2_O_2_ at a final concentration of 1 mM was supplemented in PDA medium with or without antifungal drugs. Serially diluted inoculum suspensions (2 μl) were spotted onto PDA plates and incubated at 35°C for 48 h.

### Measurement of Endogenous ROS Level

The endogenous ROS level of the AMB-resistant *A. flavus* isolate was determined by 2′,7′-dichlorofluorescin diacetate (DCF-DA) as previously described (Shekhova et al., 2017). In brief, 100 μL conidial suspensions at a concentration of 1 × 10^4^ CFU/mL were dispensed into flat-bottom 96-well plates, followed by incubation at 37°C for 18 h. After a washing step with phosphate buffered saline (PBS), the cells were stained with 10 μM DCF-DA for 30 min at 37°C in the dark. After washing with PBS, different antifungal drugs prepared in PBS were added to the cells. PBS was used as a negative control and 2 mM H_2_O_2_ was used as a positive control. The fluorescence intensity (excitation filter at 485 nm and emission filter at 530 nm) was measured by a microtiter plate reader (Infinite 200 Pro, Tecan, Switzerland) and observed under fluorescence microscope simultaneously at 37°C. The maximum fluorescence intensity observed after 2 h of incubation with drugs was recorded as a reference to the endogenous ROS level.

### Assessment of Genes Encoding Enzymes Involved in ROS Detoxification by RT-qPCR

To identify homologs of enzymes involved in ROS detoxification in *A. flavus*, the amino acid sequences of catalases (CATs) and superoxide dismutases (SODs) in *A. fumigatus* and *A. terreus* (Jukic et al., 2017) were used as queries to perform BLASTP analysis^1^ in the genome database of *A. flavus*. The primers used in Reverse transcription-quantitative PCR (RT-qPCR) were designed on the Primer3Plus^2^. The identified putative genes encoding enzymes involved in ROS detoxification in *A. flavus* and the primers are listed in Table 1.

**TABLE 1:** Primers used in this study.

Locus tag	Gene ID	Sequence (5′–3′)
**AFLA_056170**	*catA*	TGTGAAGGTCGCTACGTCTG
		ACGCTTGTAGTTCCGATGCT
**AFLA_100250**	*cat*	CGAGACACTGGCTCATTTCA
		ACCGGTGGTACTGATTCTGC
**AFLA_090690**	*cat1*	CTCCAAGCTCGTCAAGTTCC
		GATCGAAGCCAAACTTCAGC
**AFLA_122110**	*cat2*	TCAATCAGATGGAGCCTGTG
		GCCGGGTAGTAAACACTCCA
**AFLA_096210**	*cat3*	ATAATGTCGGTCGCAAGTCC
		CTTCGCATACTCTGGTGCAA
**AFLA_034380**	*cat4*	TGAGACTCTCGCCCATTTCT
		CCCAGTCCAAGTTACCCTCA
**AFLA_044930**	*sod1*	ATTGAAGGCTACGGTGTTGG
		CCCTCTTTGCTCTTCGACAC
**AFLA_068080**	*sod2*	GCGACATAAGCGGAAAACAT
		GTCTTCCTTCGCCTCTTCCT
**AFLA_033420**	*sod3*	ATGGAAATCCACCACCAAAA
		AGAGGGAGTGGTTGATGTGG
**AFLA_027580**	*sod4*	ACTCTGCCTGACCTGGCTTA
		AGTGGTGATGTCCTCCTTGG
**AFLA_088150**	*sod5*	GAGATGGCCTCCGTATTCAA
		CATCAATCCTTCCCTCTCCA
**AFLA_099000**	*sod6*	CACCAGTTCGGTGACAACAC
		GTACGGCCAAGTACGCTCTC

For assessment of expression of genes encoding enzymes involved in ROS detoxification, a total of 1 × 10^6^ CFU conidia were dispensed into *Aspergillus* minimal medium followed by incubation at 37°C for 18 h on an orbital shaker at 200 rpm. Different antifungal drugs prepared in PBS or PBS were added at 37°C for an additional 2 h on an orbital shaker at 200 rpm. Then the hyphae were harvested and total RNA was extracted following liquid nitrogen crush using TRIzol reagent (Invitrogen). cDNA was synthesized using an Advantage RT-for-PCR kit (Clontech) according to the manufacturer’s instructions. RT-qPCR was performed on an Applied Biosystems ViiA7 Real-Time PCR system using SYBR green reagent (Applied Biosystems). The cycling conditions were as follows: a 10-min initial denaturation at 95°C, followed by 40 cycles of denaturation at 95°C for 15 s, and annealing/extension at 60°C for 10 s. Changes in gene expression were calculated using the 2^–ΔΔCt^ method (Schmittgen and Livak, 2008). All experiments were performed in triplicate from biological triplicates.

### Determination of CAT, SOD and GSH-Px Activity

To determine CAT, SOD and glutathione peroxidase (GSH-Px) activity of the *A. flavus* isolate, the hyphae were harvested as conditions described in the RT-qPCR assay. The enzyme activity was determined using the CAT activity assay kit (Abcam), the SOD activity assay kit (Abcam), and GSH-Px activity assay kit (Abcam) separately according to the manufacturer’s instructions. The relative enzyme activities (%) were calculated relative to those of *A. flavus* NRRL3357 under basal conditions.

### Statistical Analysis

Experiments were performed at least three independent biological replicates. A Welch two-sample *t* test was used for significance testing of two groups. *P*-values < 0.05 were considered statistically significant.

## Results

### The AMB-Resistant Isolate of *A. flavus* Showed Sensitivity to Triazoles and Echinocandins

**FIGURE 1:**
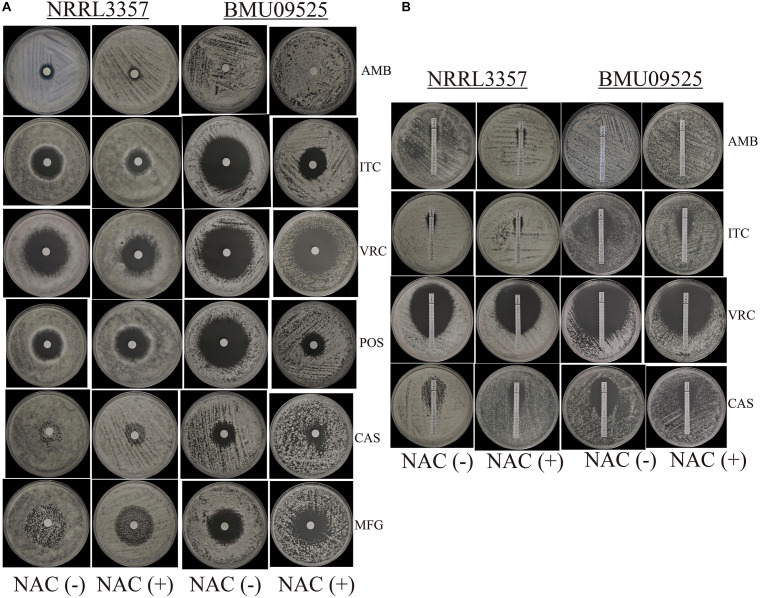
*In vitro* antifungal susceptibility testing. **(A)** *In vitro* antifungal susceptibility testing determined by disk diffusion. The antioxidant N-Acetylcysteine (NAC) was added at a concentration of 15 mM. Disks of AMB (50 μg), ITC (10 μg), VRC (5 μg), POS (5 μg), CAS (5 μg), and MFG (5 μg) were placed onto the NMH medium. Plates were incubated at 35°C for 48 h. **(B)** *In vitro* antifungal susceptibility testing determined by E-test. Plates were incubated at 35°C for 48 h.

From 117 clinical isolates of *A. flavus*, we obtained one AMB-resistant isolate of *A. flavus*, named BMU09525, with an AMB MIC of 32 μg/mL. The MICs of ITC, VRC, and POS against *A. flavus* BMU09525 were 0.06, 0.25, and 0.03 μg/mL, respectively. The MECs of CAS and MFG for *A. flavus* BMU09525 were both 0.008 μg/mL (Table 2). The results showed that the AMB-resistant isolate of *A. flavus* BMU09525 was sensitive to triazoles (ITC, VRC, POS) and echinocandins (CAS, MFG). Similar results were obtained by the disk diffusion method and E-test (Figure 1 and Table 2). Interestingly, when testing echinocandins against the *A. flavus* strain NRRL3357, microcolonies within a well-defined zone of inhibition could be seen, while testing echinocandins against AMB-resistant isolate of *A. flavus*, the inhibition ellipse of E-test strip (CAS) or the inhibition zone of the disk (CAS and MFG) was as clean as that seen in triazoles against the AMB-resistant *A. flavus* isolate, suggesting that echinocandins may exert a fungicidal effect, instead of a fungistatic effect, against the AMB-resistant isolate of *A. flavus*.

**TABLE 2:** Antifungal susceptibility testing by the broth microdilution method (μg/mL), E-test (μg/mL), and disk diffusion (mm).

	Methods	NAC	NRRL3357	BMU09525
**AMB**	**BMM**	**−**	2	32
	**E-test**	**−**	2	>32
		**+**	2	>32
	**DD**	**−**	15	0
		**+**	12	0
**ITC**	**BMM**	**−**	0.25	0.06
	**E-test**	**−**	2	0.25
		**+**	4	0.75
	**DD**	**−**	33	47
		**+**	29	34
**VRC**	**BMM**	**−**	0.5	0.25
	**E-test**	**−**	0.032	0.024
		**+**	0.064	0.064
	**DD**	**−**	40	51
		**+**	34	44
**POS**	**BMM**	**−**	0.125	0.03
	**E-test**	**−**	NA	NA
		**+**	NA	NA
	**DD**	**−**	32	46
		**+**	33	30
**CAS**	**BMM**	**−**	0.03	0.008
	**E-test**	**−**	0.25	0.064
		**+**	0.5	0.25
	**DD**	**−**	27	25
		**+**	23	22
**MFG**	**BMM**	**−**	0.03	0.008
	**E-test**	**−**	NA	NA
		**+**	NA	NA
	**DD**	**−**	40	36
		**+**	40	28

*BMM, broth microdilution method; DD, disk diffusion; NA, E-test strips were not available.*

### The AMB-Resistant Isolate of *A. flavus* Showed Hypersensitivity to Oxidative Stress, Opposite to That Observed in the AMB-Resistant *A. terreus*

**FIGURE 2:**
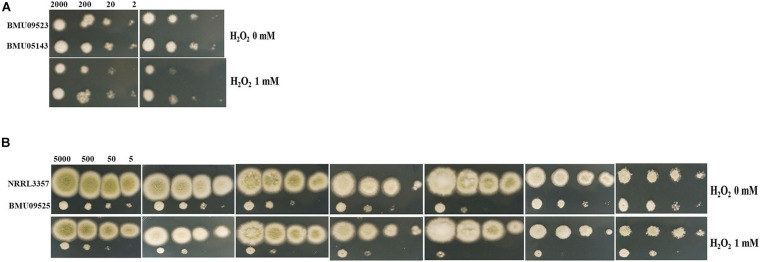
The sensitivity to H_2_O_2_ determined by spot assay. Hydrogen peroxide (H_2_O_2_) at a concentration of 1 mM was supplemented in PDA medium with or without antifungal drugs. **(A)** A total of 2, 20, 200, and 2 × 10^3^ conidia of *A. terreus* were spotted onto PDA medium, respectively. **(B)** A total of 5, 50, 500, and 5 × 10^3^ conidia of *A. flavus* were spotted onto PDA medium, respectively. Plates were incubated at 35°C and documented after 48 h.

The AMB-resistant *A. terreus* isolate showed better tolerance to H_2_O_2_ than the AMB-susceptible *A. terreus* isolates (Figure 2A), consistent with previous studies on *A. terreus*. Surprisingly, when exposed to H_2_O_2_, the AMB-resistant *A. flavus* isolate showed more obvious growth inhibition than the *A. flavus* strain NRRL3357 (Figure 2B). When H_2_O_2_ was combined with antifungals, the *A. flavus* strain NRRL3357 showed merely slight growth inhibition compared to that using antifungals alone. However, H_2_O_2_ could significantly enhance the activity of antifungals against the AMB-resistant *A. flavus* isolate (Figure 2B). The above results indicated that the AMB-resistant *A. flavus* isolate was hypersensitive to oxidative stress, in contrast to the case reported for *A. terreus*. In addition, a decreased growth rate of the AMB-resistant *A. flavus* isolate could be observed compared to the *A. flavus* strain NRRL3357 (Figure 2B).

### The AMB-Resistant Isolate of *A. flavus* Showed Elevated Basal Endogenous ROS

**FIGURE 3:**
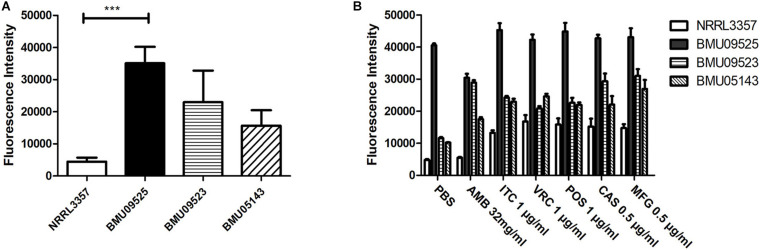
Level of endogenous ROS determined by DCF-DA assay. **(A)** The level of basal endogenous ROS. **(B)** The level of endogenous ROS induced by antifungals. Conidial were incubated at 37°C for 18 h before stained with 10 μM DCF-DA for 30 min at 37°C in the dark. After a wash step, different antifungal drugs prepared in PBS were added to the cells. The fluorescence intensity peak was observed after 2 h of incubation with drugs.

**FIGURE 4:**
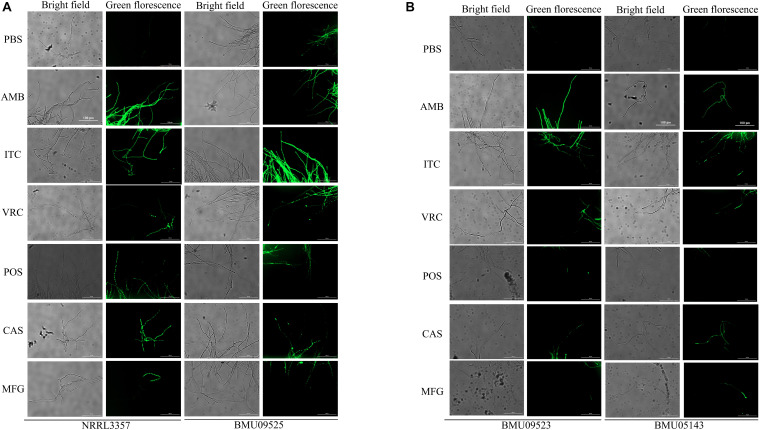
Fluorescence microscope images showing endogenous ROS in **(A)** *A. flavus*. **(B)** *A*. *terreus*. Conidial were incubated at 37°C for 18 h before stained with 10 μM DCF-DA for 30 min at 37°C in the dark. After a wash step, Fluorescence images were recorded after 2 h of incubation with drugs. AMB (32 μg/ml), ITC (1 μg/ml), VRC (1 μg/ml), POS (1 μg/ml), CAS (0.5 μg/ml), MFG (0.5 μg/ml).

The basal endogenous ROS level of the AMB-resistant *A. terreus* isolate was comparable to that of the AMB-susceptible *A. terreus* isolate, while the basal endogenous ROS level of the AMB-resistant *A. flavus* isolate was significantly higher than that of both the *A. flavus* NRRL3357 and *A. terreus* (Figures 3, 4). The elevated basal endogenous ROS in AMB-resistant *A. flavus* isolate suggested that the mechanisms of AMB resistance in AMB-resistant *A. flavus* isolate may differ from those mediating AMB resistance in *A. terreus*.

### The AMB-Resistant Isolate of *A. flavus* Showed Comparable ROS Detoxification Enzyme Activities

**FIGURE 5:**
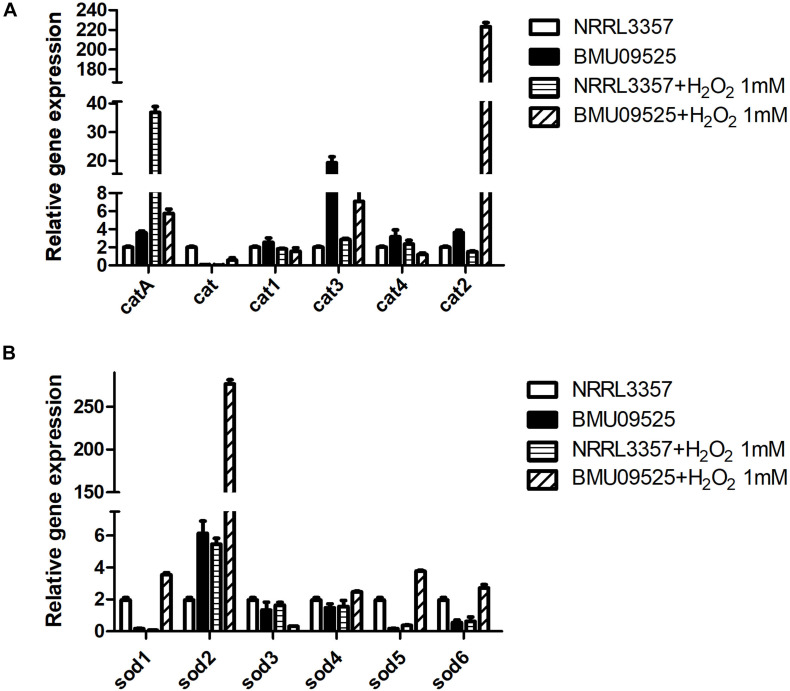
The expression level of **(A)** *cat* genes and **(B)** *sod* genes in *A. flavus*. A total of 1 × 10^6^ CFU *A. flavus* conidia were preincubated in *Aspergillus* minimal medium for 18 h at 37°C and 200 rpm before H_2_O_2_ (1 mM) was added for 2 h. Then the hyphae were harvested and total RNA was extracted following liquid nitrogen crush. Gene expression was normalized to that of beta-tubulin according to the 2^– ΔΔCt^ method. Data are presented as means ± standard deviations for three independent experiments with technical duplicates.

**FIGURE 6:**
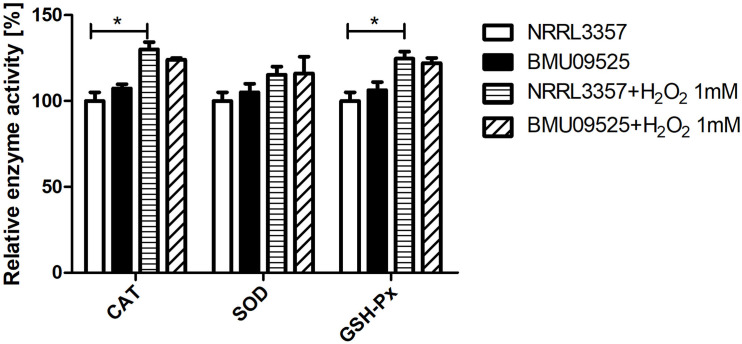
Determination of the enzyme activities of SOD, CAT and GSH-Px. A total of 1 × 10^6^ CFU *A. flavus* conidia were preincubated in *Aspergillus* minimal medium for 18 h at 37°C and 200 rpm before H_2_O_2_ (1 mM) was added for 2 h. Then the hyphae were harvested and enzyme activities were determined according to the manufacturer’s instructions. The relative enzyme activities (%) were calculated relative to those of *A. flavus* NRRL3357 under basal conditions. CAT, catalase; SOD, superoxide dismutase; GHS-Px, glutathione peroxidase.

Because the AMB-resistant *A. flavus* isolate showed elevated basal endogenous ROS level and was sensitive to oxidative stress, we further tested the expression level of *sod* and *cat* genes in *A. flavus*. A total of 6 *sod* and 6 *cat* genes were investigated in *A. flavus* (Figure 5 and Table 1). And the ROS detoxification enzyme activities, including CAT, SOD, and GSH-Px (Figure 6), were also measured.

Except for *cat* gene, the basal level of *catA*, *cat1*, *cat2*, *cat3*, and *cat4* in the AMB-resistant *A. flavus* isolate were mildly higher than that of the *A. flavus* NRRL3357. Upon H_2_O_2_ exposure, *catA*, *cat2* and *cat3* expression level increased in both the AMB-resistant *A. flavus* isolate and the *A. flavus* NRRL3357, while no significant changes of *cat*, *cat1*, and *cat4* in both two strains. However, a different picture was observed in *sod* genes expression level. In the AMB-resistant *A. flavus* isolate, only *sod2* showed a higher basal level than that of the *A. flavus* NRRL3357, while the basal expression level of *sod1*, *sod5* and *sod6* were less than that of the *A. flavus* NRRL3357. The basal expression levels of both *sod3* and *sod4* did not differ between these two *A. flavus* strains. After exposure to H_2_O_2_, *sod1*, *sod2*, *sod5*, and *sod6* showed a significant increase in the AMB-resistant *A. flavus* isolate, while only *sod2* showed elevated transcript level in the *A. flavus* NRRL3357.

Next, we tested the enzyme activities of CAT, SOD, and GSH-Px using the commercially available kits (Figure 5). The basal enzyme activities of CAT, SOD or GSH-Px in the AMB-resistant *A. flavus* isolate were not significantly different from those in the *A. flavus* NRRL3357. After exposure to H_2_O_2_, the activities of all these enzymes showed an increase but remained comparable between the two strains. Nevertheless, the increase of enzyme activities of CAT and GSH-Px in the *A. flavus* NRRL3357 was more significant than that in the AMB-resistant *A. flavus* isolate, which showed only a mild increase.

Overall, these results indicate that the basal activities of the enzyme involved in ROS detoxification in the AMB-resistant *A. flavus* isolate were comparable to those in the *A. flavus* NRRL3357, despite the different expression levels were observed. And the elevated basal endogenous ROS level in the AMB-resistant *A. flavus* isolate may not be related to abnormal ROS detoxification enzyme activities. When exposure to H_2_O_2_, however, the less remarkable increase in enzyme activities of CAT and GSH-Px in the AMB-resistant *A. flavus* isolate may result in hypersensitivity to oxidative stress.

### Triazoles and Echinocandins, Instead of AMB, Could Induce the Production of Endogenous ROS in the AMB-Resistant Isolate of *A. flavus*

To further investigate the relationships between ROS levels and antifungal susceptibilities in the AMB-resistant *A. flavus* isolate, the endogenous ROS levels induced by antifungals were determined (Figures 3, 4). With exposure to AMB, the endogenous ROS level of the AMB-susceptible *A. terreus* isolate was significantly increased, while that in the AMB-resistant *A. terreus* isolate increased slightly, consistent with the literature reports on *A. terreus*. Surprisingly, the endogenous ROS level of the *A. flavus* strain NRRL3357 increased slightly, while the endogenous ROS level of the AMB-resistant *A. flavus* isolate even showed a minor decrease despite the ROS level in the AMB-resistant *A. flavus* isolate being significantly higher than that of the *A. flavus* strain NRRL3357. With exposure to triazoles and echinocandins, the endogenous ROS levels of both *A. terreus* and *A. flavus* isolates increased to different degrees. These results strongly suggested no contribution of AMB-induced endogenous ROS to AMB resistance in the AMB-resistant *A. flavus* isolate, in contrast to the situation observed in *A. terreus*. In addition, the elevated basal endogenous ROS in the AMB-resistant *A. flavus* isolate might result in its sensitivity to triazoles and echinocandins.

### ROS Elimination by the Antioxidant NAC Decreased the Sensitivity of the AMB-Resistant *A. flavus* Isolate to Triazoles and Echinocandins

The antioxidant NAC can act as a non-specific sulfhydryl donor to scavenge intracellular ROS. Adding NAC to the medium did not show any impact on the susceptibility to AMB, corresponding to the result that no AMB-induced ROS production in the AMB-resistant *A. flavus* isolate (Figure 1A and Table 2). However, adding NAC to the medium reduced the inhibition zone of triazoles and echinocandins, indicating the decreased susceptibility of AMB-resistant *A. flavus* isolate to triazoles and echinocandins (Figure 1A and Table 2). Consistent results were obtained by E-test (Figure 1B and Table 2), which showed that NAC increased the MICs of triazoles and the MECs of echinocandins obtained from E-test strips. The above results confirmed our assumption that the increased level of endogenous ROS contributes to the sensitivity of the AMB-resistant *A. flavus* isolate to triazoles and echinocandins. Interestingly, the addition of NAC decreased the susceptibility of the *A. flavus* strain NRRL3357 to ITC, VRC, and CAS, similar to the AMB-resistant *A. flavus* isolate. Although adding NAC decreased the susceptibility of the *A. flavus* strain NRRL3357 to AMB, the addition of NAC did not change its susceptibility to POS or MFG, as no alteration in the inhibition zone diameter was observed by disk diffusion.

## Discussion

Several studies have reported that AMB can induce endogenous ROS production as its mode of action (Belenky et al., 2013; Mesa-Arango et al., 2014; Shekhova et al., 2017). Due to the intrinsic resistance of *A. terreus* to AMB, the impact of endogenous ROS production on the susceptibility of this species to AMB has been closely studied (Posch et al., 2018). Compared with AMB-susceptible *A. terreus* isolates, AMB-resistant *A. terreus* isolates presented decreased AMB-induced ROS production in mitochondria (Blatzer et al., 2015) and higher ROS-detoxifying enzyme activity (Jukic et al., 2017). However, the AMB-resistant isolate of *A. flavus* presented an elevated level of endogenous ROS regardless of exposure to AMB and was hypersensitive to oxidative stress. Further studies revealed that the less remarkable increase in enzyme activities of CAT and GSH-Px may result in its hypersensitivity to oxidative stress, compared to the observations in *A. terreus* (Blatzer et al., 2015; Jukic et al., 2017). These results suggested that the higher activity of ROS-detoxifying enzyme did not contribute to resistance to AMB in the AMB-resistant isolate of *A. flavus*. The above results indicate that the mechanisms underlying AMB resistance in AMB-resistant *A. flavus* isolate differ from those mediating AMB resistance in *A. terreus*. Although AMB resistance has not been reported to be associated with the missing ergosterol in *Aspergillus* spp. (Blum et al., 2008, 2013), the absence of ergosterol in *Candida* spp., caused by mutations in genes of the ergosterol biosynthesis (Geber et al., 1995; Sanglard et al., 2003; Martel et al., 2010a; Vincent et al., 2013; Silva et al., 2020), leading to AMB resistance and abnormal membrane structure and function, which also drastically diminished tolerance to oxidative stress (Vincent et al., 2013). These studies are consistent with the phenotypes observed in the AMB-resistant isolate of *A. flavus*. Further membrane sterol profile analysis is needed to elucidate its mechanisms of AMB resistance.

Triazoles exert antifungal effects by inhibiting sterol 14α-demethylase (CYP51A/ERG11), which prevents ergosterol biosynthesis and causes the accumulation of toxic sterols (Martel et al., 2010b; Warrilow et al., 2010, 2019). Echinocandins target the β-1,3-glucan synthase of the fungal cell wall and inhibit the synthesis of β-1,3-D glucan on the cell wall (Perlin, 2015). In addition to the above targets, several studies have reported that triazoles (Shirazi et al., 2013; Shekhova et al., 2017; Lee and Lee, 2018) and echinocandins (Belenky et al., 2013; Hao et al., 2013; Delattin et al., 2014) are capable of inducing endogenous ROS production. The AMB-resistant *A. flavus* isolate was sensitive to triazoles and echinocandins, while having elevated basal endogenous ROS. Further, H_2_O_2_ significantly enhanced the antifungal effects of triazoles and echinocandins *in vitro*, showing a synergistic effect against the AMB-resistant *A. flavus* isolate. Thus, we hypothesized that sensitivity of AMB-resistant *A. flavus* isolate to triazoles and echinocandins may be caused by elevated endogenous ROS levels. The antioxidant NAC can act as a non-specific sulfhydryl donor and is widely used to scavenge intracellular ROS (Dringen and Hamprecht, 1999; Dekhuijzen, 2004). Scavenging ROS by NAC decreased the sensitivity of AMB-resistant *A. flavus* isolate to triazoles and echinocandins confirmed our hypothesis. Interestingly, NAC did not affect the susceptibility of *A. flavus* strain NRRL3357 to POS and MFG, suggesting that ROS, albeit can be induced by POS and MFG, may not be necessary in antifungal mode of action.

Reactive oxygen species are derived from oxygen and known to be of biological importance in eukaryotic cells (Sena and Chandel, 2012). Mitochondria possess the oxidative phosphorylation system, which is the major origin of ROS generation (Zorov et al., 2014). Inappropriate electron transfer reactions in mitochondrial electron transport chain can produce excessive ROS. These highly reactive and toxic ROS can cause cellular damage, ultimately resulting in cell death (Zorov et al., 2014). Correspondingly, ROS can be eliminated by multiple antioxidant enzymes in eukaryotic cells, including SOD (Lambou et al., 2010), CAT (Shibuya et al., 2006), and GSH-Px (Margis et al., 2008). Studies in *A. terreus* (Blatzer et al., 2015; Jukic et al., 2017), the AMB-resistant *A. terreus* isolates exhibited distinct basal expression levels of *sod* and *cat* genes compared to the AMB-susceptible *A. terreus* isolates. However, the basal enzyme activities of CAT and SOD of the AMB-resistant *A. terreus* isolates were already higher than that of the AMB-susceptible *A. terreus* isolate. In this study, the two *A. flavus* strains also exhibited distinct basal expression levels of *sod* and *cat* genes. The basal expression level of *catA*, *cat3*, *cat4*, and *sod2* in the AMB-resistant *A. flavus* isolate were higher than that of the *A. flavus* NRRL3357, while the basal expression level of *cat*, *sod1*, *sod5*, and *sod6* were less than that of the *A. flavus* NRRL3357. However, the basal enzyme activities of CAT and SOD in the AMB-resistant *A. flavus* isolate were comparable to those in the *A. flavus* NRRL3357. Taken together, although no elevated enzyme activity was observed in the AMB-resistant *A. flavus* isolate in the basal condition as reported in the AMB-resistant *A. terreus*, the basal enzyme activity in the AMB-resistant *A. flavus* isolate was still comparable to the AMB-susceptible *A. terreus* isolates. Therefore, it is reasonable to speculate that the elevated basal endogenous ROS level is due to increased production rather than impaired clearance in the AMB-resistant *A. flavus* isolate. Since mitochondria are the main site of ROS production, it is likely that the elevated endogenous ROS level in the AMB-resistant *A. flavus* isolate may be caused by the dysfunction mitochondrial which may lead to overproduction of ROS. Also, the slowed growth observed in the AMB-resistant *A. flavus* isolate may be also due to mitochondrial abnormalities. However, additional studies are needed.

In conclusion, our results showed that the AMB-resistant *A. flavus* isolate presented elevated endogenous ROS levels, an opposite observation to that mediating AMB-resistance in *A. terreus*. The elevated endogenous ROS contributed to the sensitivity of the AMB-resistant *A. flavus* isolate to triazoles and echinocandins. However, further investigation is needed to elucidate the causes of elevated endogenous ROS and the resistance mechanism to AMB in *A. flavus*.

## Data Availability Statement

The original contributions presented in the study are included in the article/Supplementary material, further inquiries can be directed to the corresponding author.

## Author Contributions

TL, WC, and ZW completed the experiments. TL wrote the manuscript. XY, QW, RL, and WL revised the manuscript. WL conducted the experiments and data analysis. All authors read and approved the manuscript.

## Conflict of Interest

The authors declare that the research was conducted in the absence of any commercial or financial relationships that could be construed as a potential conflict of interest.

## Publisher’s Note

All claims expressed in this article are solely those of the authors and do not necessarily represent those of their affiliated organizations, or those of the publisher, the editors and the reviewers. Any product that may be evaluated in this article, or claim that may be made by its manufacturer, is not guaranteed or endorsed by the publisher.

## Footnotes

1
https://blast.ncbi.nlm.nih.gov/Blast.cgi

2
https://primer3plus.com/cgi-bin/dev/primer3plus.cgi

## References

[B1] Ashu E. E. Korfanty G. A. Samarasinghe H. Pum N. You M. Yamamura D. (2018). Widespread amphotericin B-resistant strains of *Aspergillus fumigatus* in Hamilton. *Canada. Infect. Drug Resist.* 11 1549–1555. 10.2147/IDR.S170952 30288065 PMC6160276

[B2] Belenky P. Camacho D. Collins J. J. (2013). Fungicidal drugs induce a common oxidative-damage cellular death pathway. *Cell. Rep.* 3 350–358. 10.1016/j.celrep.2012.12.021 23416050 PMC3656588

[B3] Blatzer M. Jukic E. Posch W. Schopf B. Binder U. Steger M. (2015). Amphotericin B Resistance in *Aspergillus terreus* Is Overpowered by Coapplication of Pro-oxidants. *Antioxid. Redox. Signal.* 23 1424–1438. 10.1089/ars.2014.6220 26054424

[B4] Blum G. Hortnagl C. Jukic E. Erbeznik T. Pumpel T. Dietrich H. (2013). New insight into amphotericin B resistance in *Aspergillus terreus*. *Antimicrob. Agents Chemother.* 57 1583–1588. 10.1128/AAC.01283-12 23318794 PMC3623369

[B5] Blum G. Perkhofer S. Haas H. Schrettl M. Wurzner R. Dierich M. P. (2008). Potential basis for amphotericin B resistance in *Aspergillus terreus*. *Antimicrob. Agents Chemother.* 52 1553–1555. 10.1128/AAC.01280-07 18268082 PMC2292529

[B6] Brown G. D. Denning D. W. Gow N. A. Levitz S. M. Netea M. G. White T. C. (2012). Hidden killers: human fungal infections. *Sci. Transl. Med.* 4:165rv113. 10.1126/scitranslmed.3004404 23253612

[B7] Clinical and Laboratory Standards Institute (CLSI). (2017). *Reference Method for Broth Dilution Antifungal Susceptibility Testing of Filamentous Fungi. CLSI Standard M38*, 3rd Edn. Wayne: Clinical and Laboratory Standards Institute.

[B8] Dekhuijzen P. N. (2004). Antioxidant properties of N-acetylcysteine: their relevance in relation to chronic obstructive pulmonary disease. *Eur. Respir. J.* 23 629–636. 10.1183/09031936.04.00016804 15083766

[B9] Delattin N. Cammue B. P. Thevissen K. (2014). Reactive oxygen species-inducing antifungal agents and their activity against fungal biofilms. *Future Med. Chem.* 6 77–90. 10.4155/fmc.13.189 24358949

[B10] Dringen R. Hamprecht B. (1999). N-acetylcysteine, but not methionine or 2-oxothiazolidine-4-carboxylate, serves as cysteine donor for the synthesis of glutathione in cultured neurons derived from embryonal rat brain. *Neurosci. Lett.* 259 79–82. 10.1016/s0304-3940(98)00894-5 10025562

[B11] Geber A. Hitchcock C. A. Swartz J. E. Pullen F. S. Marsden K. E. Kwon-Chung K. J. (1995). Deletion of the *Candida glabrata ERG3* and *ERG11* genes: effect on cell viability, cell growth, sterol composition, and antifungal susceptibility. *Antimicrob. Agents Chemother.* 39 2708–2717. 10.1128/aac.39.12.2708 8593007 PMC163017

[B12] Gray K. C. Palacios D. S. Dailey I. Endo M. M. Uno B. E. Wilcock B. C. (2012). Amphotericin primarily kills yeast by simply binding ergosterol. *Proc. Natl. Acad. Sci. U. S. A.* 109 2234–2239. 10.1073/pnas.1117280109 22308411 PMC3289339

[B13] Grazziotin L. R. Moreira L. B. Ferreira M. A. P. (2018). Comparative Effectiveness and Safety between Amphotericin B Lipid-Formulations: a Systematic Review. *Int. J. Technol. Assess. Health Care* 34 343–351. 10.1017/S026646231800034X 29897025

[B14] Hadrich I. Makni F. Neji S. Cheikhrouhou F. Bellaaj H. Elloumi M. (2012). Amphotericin B *in vitro* resistance is associated with fatal *Aspergillus flavus* infection. *Med. Mycol.* 50 829–834. 10.3109/13693786.2012.684154 22587728

[B15] Hao B. Cheng S. Clancy C. J. Nguyen M. H. (2013). Caspofungin kills *Candida albicans* by causing both cellular apoptosis and necrosis. *Antimicrob. Agents Chemother.* 57 326–332. 10.1128/AAC.01366-12 23114781 PMC3535936

[B16] Jukic E. Blatzer M. Posch W. Steger M. Binder U. Lass-Florl C. (2017). Oxidative Stress Response Tips the Balance in *Aspergillus terreus* Amphotericin B Resistance. *Antimicrob. Agents Chemother.* 61:e00670–17. 10.1128/AAC.00670-17 28739793 PMC5610508

[B17] Kristanc L. Bozic B. Jokhadar S. Z. Dolenc M. S. Gomiscek G. (2019). The pore-forming action of polyenes: from model membranes to living organisms. *Biochim. Biophys. Acta Biomembr.* 1861 418–430. 10.1016/j.bbamem.2018.11.006 30458121

[B18] Lambou K. Lamarre C. Beau R. Dufour N. Latge J. P. (2010). Functional analysis of the superoxide dismutase family in *Aspergillus fumigatus*. *Mol. Microbiol.* 75 910–923. 10.1111/j.1365-2958.2009.07024.x 20487287

[B19] Lee W. Lee D. G. (2018). Reactive oxygen species modulate itraconazole-induced apoptosis via mitochondrial disruption in *Candida albicans*. *Free Radic. Res.* 52 39–50. 10.1080/10715762.2017.1407412 29157011

[B20] Margis R. Dunand C. Teixeira F. K. Margis-Pinheiro M. (2008). Glutathione peroxidase family - an evolutionary overview. *FEBS J.* 275 3959–3970. 10.1111/j.1742-4658.2008.06542.x 18616466

[B21] Martel C. M. Parker J. E. Bader O. Weig M. Gross U. Warrilow A. G. (2010a). A clinical isolate of *Candida albicans* with mutations in ERG11 (encoding sterol 14alpha-demethylase) and ERG5 (encoding C22 desaturase) is cross resistant to azoles and amphotericin B. *Antimicrob. Agents Chemother.* 54 3578–3583. 10.1128/AAC.00303-10 20547793 PMC2934972

[B22] Martel C. M. Parker J. E. Warrilow A. G. Rolley N. J. Kelly S. L. Kelly D. E. (2010b). Complementation of a *Saccharomyces cerevisiae* ERG11/CYP51 (sterol 14alpha-demethylase) doxycycline-regulated mutant and screening of the azole sensitivity of *Aspergillus fumigatus* isoenzymes CYP51A and CYP51B. *Antimicrob. Agents Chemother.* 54 4920–4923. 10.1128/AAC.00349-10 20733045 PMC2976139

[B23] Mesa-Arango A. C. Trevijano-Contador N. Roman E. Sanchez-Fresneda R. Casas C. Herrero E. (2014). The production of reactive oxygen species is a universal action mechanism of Amphotericin B against pathogenic yeasts and contributes to the fungicidal effect of this drug. *Antimicrob. Agents Chemother.* 58 6627–6638. 10.1128/AAC.03570-14 25155595 PMC4249417

[B24] Perlin D. S. (2015). Mechanisms of echinocandin antifungal drug resistance. *Ann. N. Y. Acad. Sci.* 1354 1–11. 10.1111/nyas.12831 26190298 PMC4626328

[B25] Perlin D. S. Rautemaa-Richardson R. Alastruey-Izquierdo A. (2017). The global problem of antifungal resistance: prevalence, mechanisms, and management. *Lancet Infect. Dis.* 17 e383–e392. 10.1016/S1473-3099(17)30316-X 28774698

[B26] Posch W. Blatzer M. Wilflingseder D. Lass-Florl C. (2018). *Aspergillus terreus*: novel lessons learned on amphotericin B resistance. *Med. Mycol.* 56 73–82. 10.1093/mmy/myx119 29538736

[B27] Qiao J. Kontoyiannis D. P. Wan Z. Li R. Liu W. (2007). Antifungal activity of statins against *Aspergillus* species. *Med. Mycol.* 45 589–593. 10.1080/13693780701397673 18033614

[B28] Rudramurthy S. M. Paul R. A. Chakrabarti A. Mouton J. W. Meis J. F. (2019). Invasive Aspergillosis by *Aspergillus flavus*: epidemiology, Diagnosis, Antifungal Resistance, and Management. *J. Fungi.* 5:55. 10.3390/jof5030055 31266196 PMC6787648

[B29] Sanglard D. Ischer F. Parkinson T. Falconer D. Bille J. (2003). *Candida albicans* mutations in the ergosterol biosynthetic pathway and resistance to several antifungal agents. *Antimicrob. Agents Chemother.* 47 2404–2412. 10.1128/aac.47.8.2404-2412.2003 12878497 PMC166068

[B30] Schmittgen T. D. Livak K. J. (2008). Analyzing real-time PCR data by the comparative C(T) method. *Nat. Protoc.* 3 1101–1108. 10.1038/nprot.2008.73 18546601

[B31] Sena L. A. Chandel N. S. (2012). Physiological roles of mitochondrial reactive oxygen species. *Mol. Cell.* 48 158–167. 10.1016/j.molcel.2012.09.025 23102266 PMC3484374

[B32] Shekhova E. Kniemeyer O. Brakhage A. A. (2017). Induction of Mitochondrial Reactive Oxygen Species Production by Itraconazole, Terbinafine, and Amphotericin B as a Mode of Action against *Aspergillus fumigatus*. *Antimicrob. Agents Chemother.* 61:e00978–17. 10.1128/AAC.00978-17 28848005 PMC5655112

[B33] Shibuya K. Paris S. Ando T. Nakayama H. Hatori T. Latge J. P. (2006). Catalases of *Aspergillus fumigatus* and inflammation in aspergillosis. *Nihon. Ishinkin. Gakkai. Zasshi.* 47 249–255. 10.3314/jjmm.47.249 17086155

[B34] Shirazi F. Pontikos M. A. Walsh T. J. Albert N. Lewis R. E. Kontoyiannis D. P. (2013). Hyperthermia sensitizes *Rhizopus oryzae* to posaconazole and itraconazole action through apoptosis. *Antimicrob. Agents Chemother.* 57 4360–4368. 10.1128/AAC.00571-13 23817366 PMC3754336

[B35] Silva L. N. Oliveira S. S. C. Magalhaes L. B. Andrade Neto V. V. Torres-Santos E. C. Carvalho M. D. C. (2020). Unmasking the Amphotericin B Resistance Mechanisms in *Candida haemulonii* Species Complex. *ACS Infect. Dis.* 6 1273–1282. 10.1021/acsinfecdis.0c00117 32239912

[B36] Stone N. R. Bicanic T. Salim R. Hope W. (2016). Liposomal Amphotericin B (AmBisome(^®^)): a Review of the Pharmacokinetics, Pharmacodynamics, Clinical Experience and Future Directions. *Drugs* 76 485–500. 10.1007/s40265-016-0538-7 26818726 PMC4856207

[B37] Vaezi A. Fakhim H. Arastehfar A. Shokohi T. Hedayati M. T. Khodavaisy S. (2018). *In vitro* antifungal activity of amphotericin B and 11 comparators against *Aspergillus terreus* species complex. *Mycoses* 61 134–142. 10.1111/myc.12716 29064123

[B38] Vincent B. M. Lancaster A. K. Scherz-Shouval R. Whitesell L. Lindquist S. (2013). Fitness trade-offs restrict the evolution of resistance to amphotericin B. *PLoS Biol.* 11:e1001692. 10.1371/journal.pbio.1001692 24204207 PMC3812114

[B39] Warrilow A. G. Melo N. Martel C. M. Parker J. E. Nes W. D. Kelly S. L. (2010). Expression, purification, and characterization of *Aspergillus fumigatus* sterol 14-alpha demethylase (CYP51) isoenzymes A and B. *Antimicrob. Agents Chemother.* 54 4225–4234. 10.1128/AAC.00316-10 20660663 PMC2944604

[B40] Warrilow A. G. S. Parker J. E. Price C. L. Rolley N. J. Nes W. D. Kelly D. E. (2019). Isavuconazole and voriconazole inhibition of sterol 14alpha-demethylases (CYP51) from *Aspergillus fumigatus* and *Homo sapiens*. *Int. J. Antimicrob. Agents* 54 449–455. 10.1016/j.ijantimicag.2019.07.011 31310805

[B41] Zorov D. B. Juhaszova M. Sollott S. J. (2014). Mitochondrial reactive oxygen species (ROS) and ROS-induced ROS release. *Physiol. Rev.* 94 909–950. 10.1152/physrev.00026.2013 24987008 PMC4101632

